# MiR-769-5p of macrophage exosomes induced by GRP78 promotes stemness and chemoresistance in colorectal cancer

**DOI:** 10.1038/s41419-025-07466-7

**Published:** 2025-03-05

**Authors:** Jinmiao Tian, Lichao Zhang, Xiaoqin La, Xiaxia Fan, Zhuoyu Li

**Affiliations:** 1 Key Laboratory of Chemical Biology and Molecular Engineering of the National Ministry of Education, Institute of Biotechnology, Shanxi University, Taiyuan, 030006 China; 2 Institutes of Biomedical Sciences, Shanxi University, Taiyuan, 030006 China

**Keywords:** Cancer microenvironment, Tumour biomarkers

## Abstract

The tumor microenvironment (TME) plays an important role in tumorigenesis and development. Tumor-associated macrophages (TAMs) are essential members of the TME, the exosomes and miRNAs they secrete are crucial in tumor regulation. Our previous study showed that GRP78-induced macrophages infinitely tend to be M2-type TAMs. In this study, the exosomes of M0 and GRP78-induced macrophage were collected and co-incubated with colorectal cancer (CRC) cells. The results implied that macrophage exosomes induced by GRP78 (GRP78-exos) significantly promoted stemness and chemoresistance in CRC in vitro and in vivo. Further, the top 5 miRNAs upregulated in GRP78-exos were obtained from miRNA sequencing data. The qRT-PCR validation revealed that miR-769-5p was the most observably upregulated and could be directly transferred into CRC cells via GRP78-exos. Mechanistically, the study indicated that miR-769-5p targeted MAPK1 to regulate the cell cycle-related proteins RB1, cyclin D1, and cyclin E1. This contributes to CRC cells entering a quiescent state, which leads to the development of chemoresistance. Moreover, miR-769-5p is also expressed higher in the tissues of 5-FU-resistant CRC patients. In summary, the findings indicate a novel function of miR-769-5p as a potential marker for the diagnosis and treatment of chemotherapy resistance in CRC.

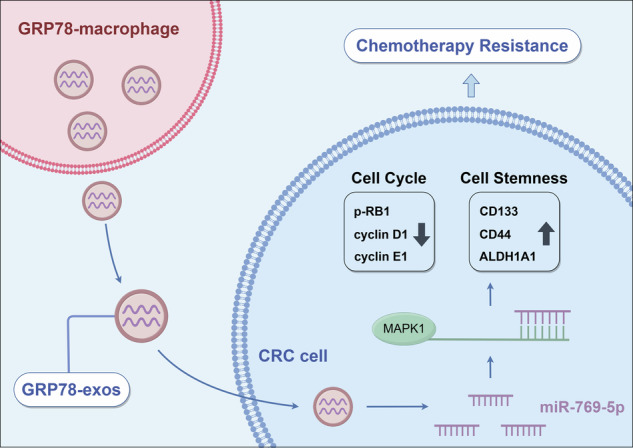

## Introduction

Colorectal cancer (CRC) is one of the common gastrointestinal malignancies. In the late 1990s, CRC was only the fourth leading cause of cancer deaths, but in recent years it has been ranked number one in men and number two in women [1]. Despite the emergence of many novel therapies, such as targeted therapy and immunotherapy, chemotherapeutic agents represented by 5-fluorouracil (5-FU) are still one of the main treatments for CRC patients [2]. During the initial treatment phase, patients generally respond well to chemotherapeutic drugs, but the majority of patients gradually develop drug resistance, and more than 40% of CRC patients eventually die from recurrence and metastasis [3]. Current research shows that cancer stem cells (CSCs) are the fundamental factor of chemotherapy resistance, which is a small number of cells with self-renewal ability. Thus, inhibiting the stemness of cancer cells is an essential direction to solve the challenge of chemotherapy resistance [4–6].

Cancer is not just a disease, but a complex ecosystem involving a wide range of non-cancerous cells and their myriad interactions within the tumor. Cancer development and progression are intricately linked to the tumor microenvironment (TME). TME includes a variety of immune cells, cancer-associated fibroblasts, endothelial cells, pericytes, and various other cells [7]. These cells were once thought to be bystanders to tumorigenesis, but are now known to play a key role in cancer pathogenesis and therapeutic response [8]. Tumor-associated macrophages (TAMs) are the most numerous immune cells in the TME [9], mainly presenting the M2 type, and communicate intercellularly with tumor cells through the release of cytokines, chemokines, growth factors, proteases, and other paracrine signals to form an immune-suppressive microenvironment that helps tumor cells to resist treatment [10, 11]. Our previous study showed that glucose-regulated protein 78 (GRP78) secreted by CRC cells induces macrophage M2 polarization by promoting lipolysis [12]. However, it is not clear how GRP78-induced M2-like macrophages communicate with tumor cells and regulate stemness and chemotherapy resistance.

One of the main modes of long-distance intercellular communication is extracellular vesicles. Exosomes are the focus of research on extracellular vesicles. Exosomes are tiny vesicles with a lipid bilayer, which can transmit biological information between cells and play an essential role in intercellular communication and the maintenance of homeostasis in the internal environment due to the rich content they contain [13, 14]. It can also serve as an important mediator of communication between tumor cells and stromal cells in the TME, and is involved in a variety of tumor-promoting activities [15]. It has been shown that M2 macrophage-derived exosomes promote tumor angiogenesis [16], metastasis [17] and maintain cancer cell stemness [18, 19].

MicroRNA (miRNA) is one of the important contents of exosomes, accounting for a relatively large proportion of the nucleic acid fraction. MiRNAs are a kind of highly conserved short-stranded non-coding RNAs that inhibit the expression of target genes by binding to the 3’-UTR region of the corresponding mRNA [20], and regulate many physiological processes such as cell proliferation, invasion, and migration. Therefore, miRNA plays an important role in the intercellular communication mediated by exosomes, which is a hot topic in the study of tumor microenvironment. However, the mechanism by which miRNAs in GRP78-induced macrophage-derived exosomes regulate tumor-associated target genes remains to be investigated.

In this study, we reveal that miR-769-5p is transferred from M2-like macrophages induced by GRP78 into CRC cells via exosomes, which in turn promotes stemness and chemotherapy resistance in CRC. Furthermore, miR-769-5p targets and inhibits MAPK1 in CRC cells, which blocks the cell cycle and induces CRC cells to enter the quiescent phase, thereby promoting stemness and chemotherapy resistance. Moreover, high expression of miR-769-5p is closely associated with the development of chemotherapy resistance in CRC. This suggests that miR-769-5p has the potential for development as a novel marker for the diagnosis of CRC and as a target for the treatment of drug resistance in CRC.

## Materials and methods

### Colorectal cancer (CRC) cDNA microarray

The CRC cDNA microarray (HColA030CS02 and HColA060CS02) was purchased from Shanghai Outdo Biotech Company (Shanghai, China) with 40 CRC tissues and paired para-cancer tissues (Ethics No. SHYJS-CP-1701015 and No. SHYJS-CP-1701017).

### Data analysis for single-cell sequencing

In this study, single-cell sequencing analysis was performed based on GSE132465 from the GEO database, and single-cell quality control, normalization, and clustering operations were performed using Seurat V5 [21]. For each sample, cells expressing less than 200 genes and more than 4000 genes, with UMI count greater than 20,000 and with more than 15% mitochondrial genes were excluded (Fig. S1A–E). Sample integration was performed using the CCAIntegration method in the IntegrateLayers function. Subsequently, cell clustering was performed using the tSNE method, the marker genes for each cluster of cells were calculated using the FindAllMarkers function, and cell-assisted annotation was performed in the ACT database. Cell-to-cell communication analysis was subsequently performed using the CellChat package [22].

### Isolation of exosomes from cell supernatant

Exosomes were extracted from cell supernatant using ultracentrifugation. Human monocytes THP-1 were treated with a final concentration of 150 nM of PMA for 12 h to induce M0 macrophages. M0 macrophages were then treated with purified GRP78 protein for 24 h. Both M0 macrophages and GRP78-induced macrophages were cultured in a serum-free medium for 48 h. The cell supernatants were collected. They were centrifuged at 300 × *g,* 4 °C for 10 min to remove cellular debris. The supernatants were centrifuged again at 3000 × *g*, 4 °C for 15 min to remove other impurities. The supernatants were collected by centrifugation at 12,000 × *g* for 70 min and the exosomes were washed with sterile PBS. The exosomes were purified by centrifugation again at 12,000 × *g* for 70 min. Afterwards, exosomes were resuspended with PBS and filtered through a 0.22 μm filter membrane. The total protein concentration of exosomes was determined by the BCA method.

### Statistical methods

Data were analyzed statistically using GraphPad Prism 8. The data are presented as mean values ± standard deviation (SD). Unpaired two-tailed *t*-test was used to compare data between two groups and one-way analysis of variance (ANOVA) was used for multiple comparisons. *p* < 0.05 was considered statistically significant.

## Result

### Macrophages induced by GRP78 promote chemoresistance and stemness in CRC cells

**Fig. 1:**
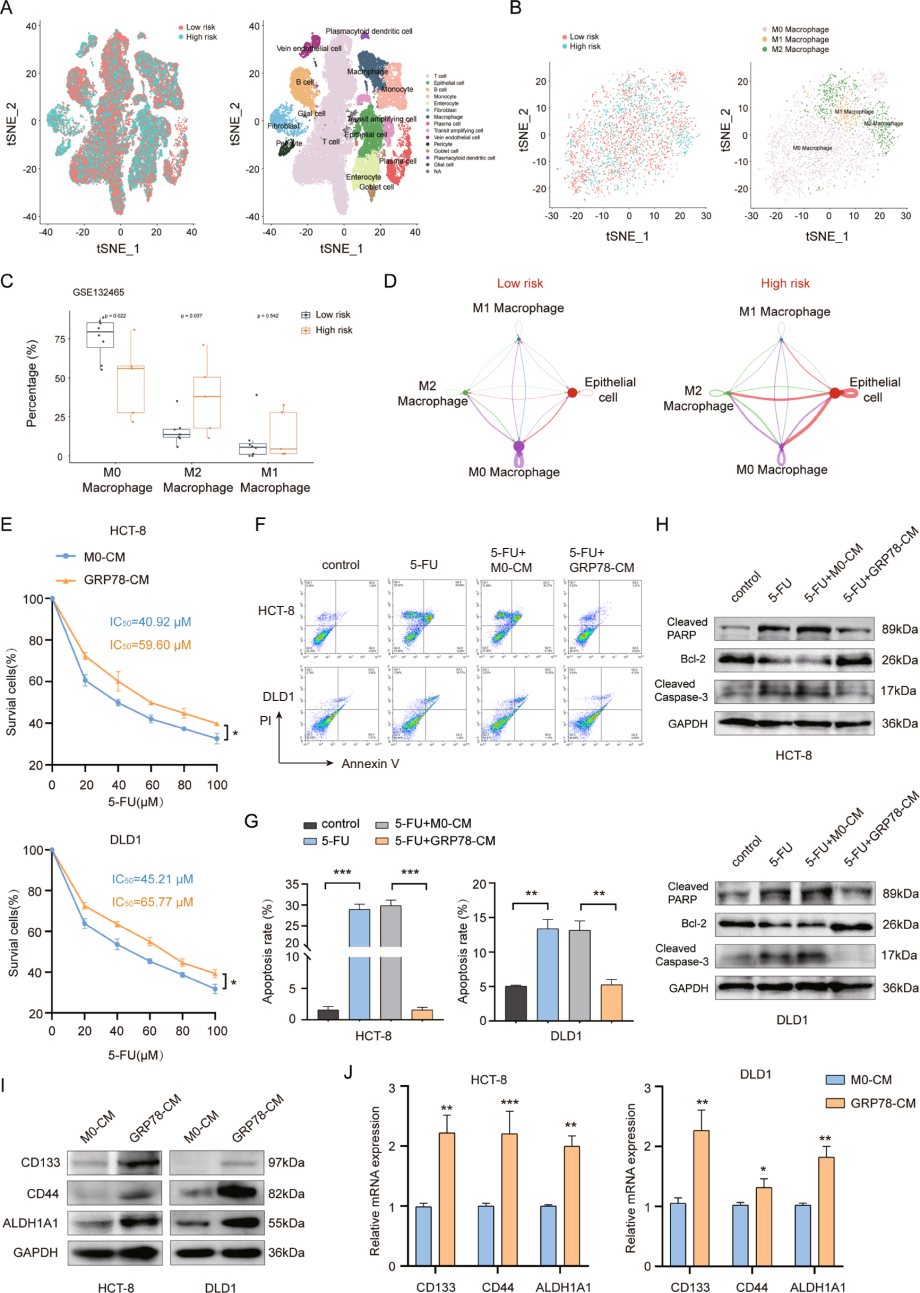
GRP78-induced macrophages promote CRC chemoresistance and enhance tumor cell stemness. **A** The tSNE clustering plot for single cells. **B** The tSNE clustering plot for macrophage subsets. **C** Box plot of enrichment of M0, M1, and M2 macrophages in high and low-risk groups for 5-FU resistance. **D** Diagram of a cellular communication network. The size of the dots in the diagram represents the relative number of cells, and the thickness of the connecting lines represents the strength of intercellular communication. **E** The effects of M0-CM and GRP78-CM on the viability of HCT-8 and DLD1 cells were detected by MTT and IC_50_ values were calculated. **F** The effects of M0-CM and GRP78-CM on 5-FU-induced apoptosis in HCT-8 and DLD1 cells were detected by flow cytometry. **G** Statistical plots of apoptosis rates of HCT-8 and DLD1 cells for (**F**). **H** The effects of M0-CM and GRP78-CM on the expression of pro-apoptotic proteins cleaved-PARP and cleaved-caspase 3 and anti-apoptotic protein Bcl-2 in the presence of 5-FU by western blot. **I** The effects of M0-CM and GRP78-CM on the expression of HCT-8 and DLD1 cell stemness markers CD133, CD44 and ALDH1A1 proteins by western blot. **J** The effects of M0-CM and GRP78-CM on the expression of stemness markers CD133, CD44 and ALDH1A1 mRNA in HCT-8 and DLD1 cells as detected by qRT-PCR. Data are expressed as SD ± mean, *n* = 3. *p < 0.05, **p < 0.01, ***p < 0.001.

Our previous study found that GRP78 treatment induced M2 polarization of macrophages, thereby promoting tumor progression [12]. To further determine the relationship between M2 macrophages and tumor chemoresistance, we analyzed the publicly available CRC single-cell sequencing dataset (GSE132465). Fourteen cell types were annotated by t-SNE clustering (Fig. 1A). Then we further annotated macrophages based on the characterized genes and obtained M0, M1, and M2 macrophages (Fig. 1B). Next, we utilized the prognostic model constructed by the 5-FU resistance gene to classify the samples in the single-cell dataset into a low-risk group and a high-risk group for chemotherapy resistance [23]. The analysis results showed that M2 macrophages were significantly enriched in the high-risk group for chemotherapy resistance (Fig. 1C), indicating a strong correlation between M2 macrophages and CRC chemotherapy resistance. In addition, cell communication analysis showed that the communication between M2 macrophages and tumor epithelial cells was significantly increased in the high-risk group (Fig. 1D). This implied that M2 macrophages might be involved in tumor progression by secreting signaling. Therefore, based on previous studies, we here explored whether M2-like macrophages induced by GRP78 modulate CRC chemoresistance.

To investigate the effect of GRP78-induced macrophages on CRC chemoresistance, we collected conditioned medium of M0 macrophages (M0-CM) and GRP78-induced macrophage conditioned medium (GRP78-CM). The results of MTT showed that GRP78-CM treatment significantly elevated the IC_50_ values of chemotherapeutic drug 5-FU on HCT-8 and DLD1 cells (Fig. 1E). The results of the flow cytometry assay revealed that GRP78-CM treatment relieved the inhibition of 5-FU on CRC cells and significantly reduced the apoptosis rate of CRC cells (Fig. 1F, G). In addition, western blot results also showed that GRP78-CM treatment inhibited the expression of pro-apoptotic proteins cleaved-PARP and cleaved-caspase 3, and promoted the expression of anti-apoptotic protein Bcl-2 (Fig. 1H). The enhancement of tumor cell stemness is often regarded as the fundamental factor in the development of chemotherapy resistance. We next examined the effects of M0-CM and GRP78-CM on the stemness of CRC cells. CD133, CD44, and ALDH1A1 are classical markers of CRC stem cells. qRT-PCR and western blot results showed that GRP78-CM treatment markedly enhanced the mRNA and protein expression levels of these three markers (Fig. 1I, J). Taken together, GRP78-CM promoted CRC chemotherapy resistance and enhanced its stemness.

### Exosomes from GRP78-induced macrophage modulate chemoresistance and stemness in CRC cells

**Fig. 2:**
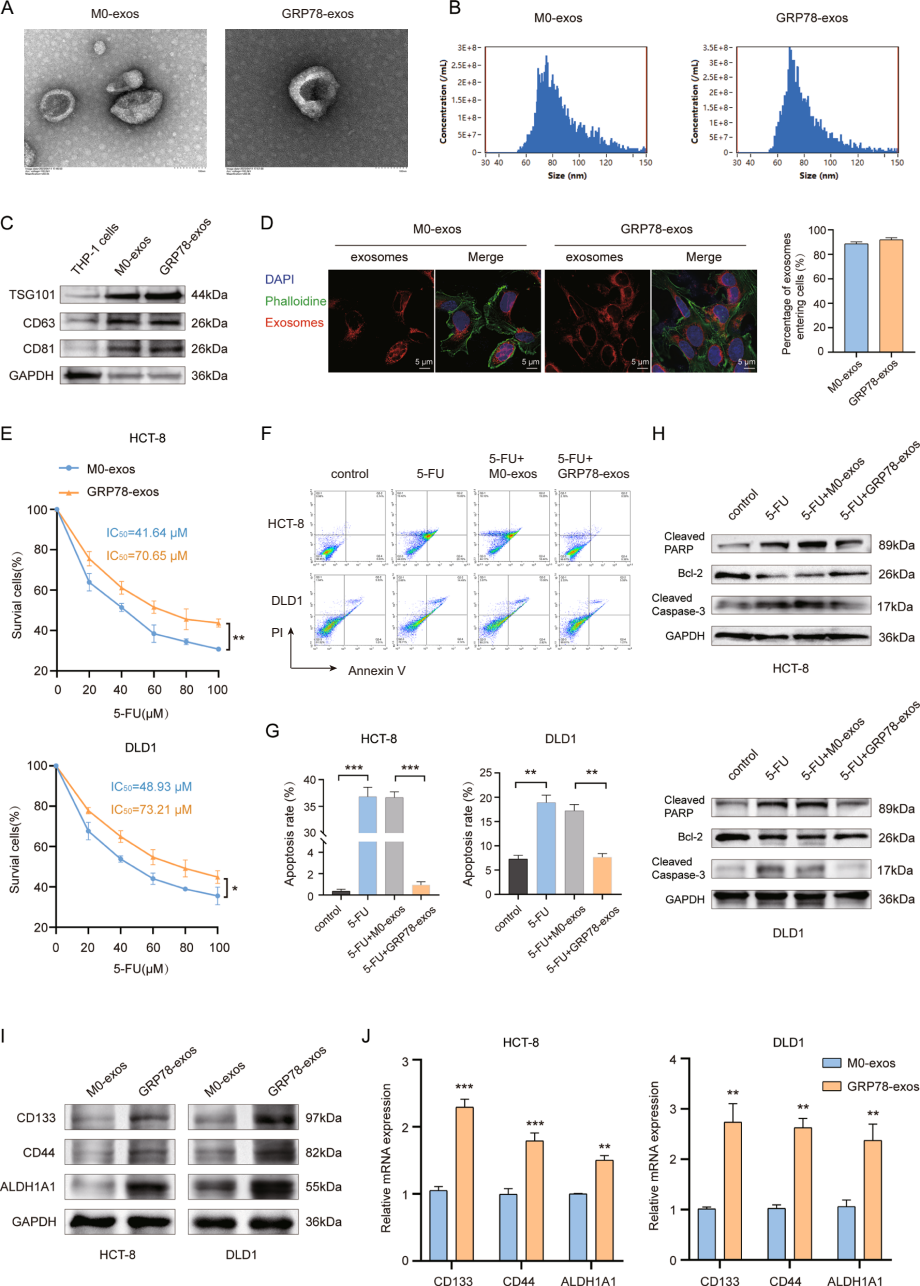
GRP78-induced macrophage-derived exosomes modulate chemoresistance and stemness in CRC cells. **A** Transmission electron microscope (TEM) micrographs of M0-exos and GRP78-exos. **B** Measurement of particle size of M0-exos and GRP78-exos by nanoparticle tracking analysis (NTA). **C** Expression of exosome marker proteins TSG101, CD63, and CD81 in THP-1 cells, M0-exos and GRP78-exos was detected by western blot. THP-1 cells served as a control. **D** Internalization of M0-exos and GRP78-exos was observed by fluorescence microscopy, and the proportion of them entering cells was counted. DAPI: nucleus. Phalloidin: representing cytoskeleton. Scale bar: 5 μm. **E** The effects of M0-exos and GRP78-exos on the viability of HCT-8 and DLD1 cells were detected by MTT and IC_50_ values were calculated. **F** The effects of M0-exos and GRP78-exos on 5-FU-induced apoptosis in HCT-8 and DLD1 cells were detected by flow cytometry. **G** Statistical plots of apoptosis rates of HCT-8 and DLD1 cells for (**F**). **H** The effects of M0-exos and GRP78-exos on the expression of pro-apoptotic proteins cleaved-PARP and cleaved-caspase 3 and anti-apoptotic protein Bcl-2 in the presence of 5-FU by western blot. **I** The effects of M0-exos and GRP78-exos on the protein expression of stemness markers CD133, CD44 and ALDH1A1 in HCT-8 and DLD1 cells were examined by western blot. **J** The effects of M0-exos and GRP78-exos on the expression of stemness markers CD133, CD44 and ALDH1A1 mRNA in HCT-8 and DLD1 cells were detected by qRT-PCR. Data are expressed as SD ± mean, *n* = 3. *p < 0.05, **p < 0.01, ***p < 0.001.

Exosomes play an indispensable role in the regulation of tumors [24, 25]. Therefore, we speculated that the component playing a key role in GRP78-CM might be exosomes. We used ultracentrifugation to respectively extract exosomes from M0 and GRP78-induced macrophages and characterized them. Transmission electron microscopy (TEM) observation showed a double-layer membrane structure, which is a hallmark of exosomes (Fig. 2A), and nanoparticle tracking analysis (NTA) showed particle sizes between 50–150 nm, which is consistent with exosome characteristics (Fig. 2B). In addition, the classical exosome markers TSG101, CD63, and CD81 were detected in these vesicles (Fig. 2C). Afterwards, exosomes secreted by M0 macrophages (M0-exos) and GRP78-induced macrophages (GRP78-exos) were labeled with fluorescent dyes, and then, were added to the culture medium of CRC cells to track their cell localization. Microscopic observation showed that the exosomes with red fluorescence entered into CRC cells labeled with green fluorescence (Fig. 2D). This result suggests that M0-exos and GRP78-exos enter and function in CRC cells.

We further evaluated the roles of M0-exos and GRP78-exos in chemoresistance and stemness of CRC cells. The results of MTT showed that GRP78-exos treatment significantly increased the IC_50_ values of 5-FU in HCT-8 and DLD1 cells compared with M0-exos (Fig. 2E). Flow cytometry and western blot results also showed that GRP78-exos treatment weakened the killing effect of 5-FU on CRC cells (Fig. 2F–H), suggesting that GRP78-exos promoted the tolerance of CRC cells to 5-FU. In addition, qRT-PCR and western blot results showed that GRP78-exos treatment dramatically upregulated the expression of stem cell markers CD133, CD44, and ALDH1A1 in CRC cells (Fig. 2I, J). These results suggest that GRP78-induced macrophage-derived exosomes promote tolerance to chemotherapeutic drugs and enhance the stemness of CRC cells.

### GRP78-induced macrophage exosomes promote chemotherapy resistance in a mouse model

**Fig. 3:**
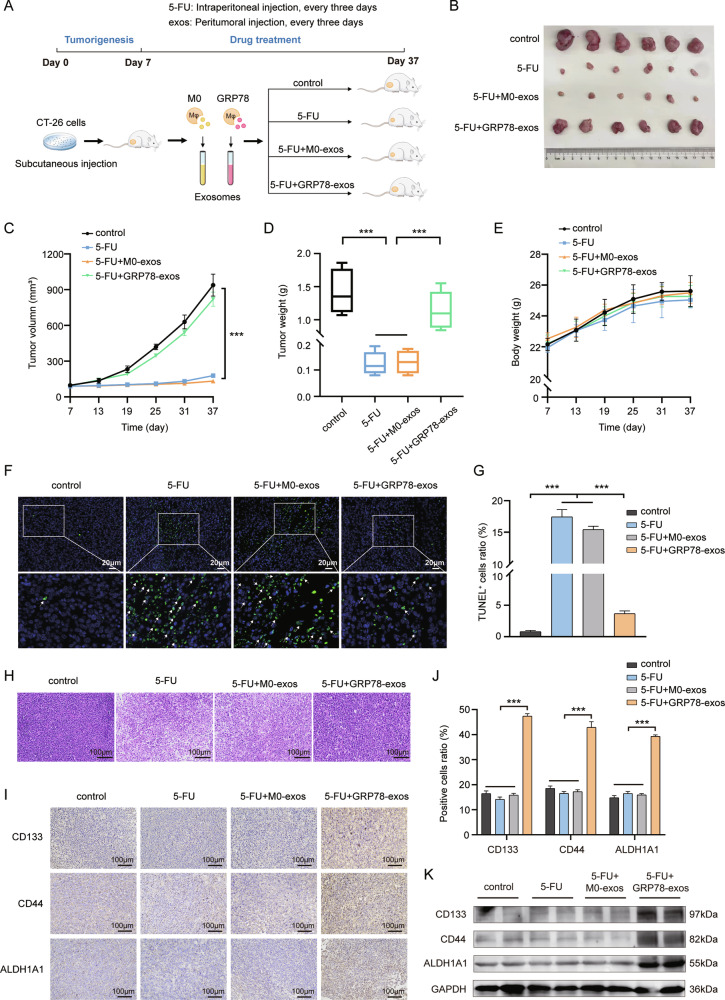
GRP78-induced macrophage-derived exosomes promote chemotherapy resistance in a mouse model. **A** Experimental design diagram of the mouse model. **B** Images of mouse tumors. **C** Graph of tumor volume changes in mice from 7–37 days. Data are expressed as SD ± mean, *n* = 6 mice. **D** Statistical plot of tumor weights in mice. **E** Graph of body weight changes in mice from 7–37 days. **F** Representative images of TUNEL staining of mouse tumor tissues. White arrows represent TUNEL-positive cells. Scale bar: 20 μm. **G** Statistical graph of TUNEL-positive cells in mouse tumor tissues. **H** Representative images of HE staining of mouse tumor tissues. Scale bar: 100 μm. **I** Representative images of CD133, CD44, ALDH1A1 immunohistochemical staining of mouse tumor tissues. Scale bar: 100 μm. **J** Statistical graph of positive cells in immunohistochemical staining for (**I**). **K** The protein expression of tumor tissue stemness markers CD133, CD44, and ALDH1A1 was detected by western blot in each group of mice. Data are expressed as SD ± mean, ***p < 0.001.

Next, we further explored the role of GRP78-exos in vivo. In tumor-bearing mice, 5-FU, M0-exos, or GRP78-exos were concurrently administered, and the volume of the mice’s tumors was measured weekly (Fig. 3A). The results showed that 5-FU treatment greatly inhibited tumor growth in mice compared with controls, but the volume and weight of the tumor were significantly increased in mice given GRP78-exos concurrently (Fig. 3B–D). All treatments had no obvious effect on the body weight of mice (Fig. 3E). Next, we performed TUNEL staining of the tumor tissues of mice in each group, which showed that green fluorescence was notably increased in the 5-FU group and the 5-FU + M0-exos group compared with the control group, but decreased in the 5-FU + GRP78-exos group, suggesting that the treatment of GRP78-exos caused a decrease in TUNEL-positive cells and suppressed the killing effect of 5-FU on CRC cells (Fig. 3F, G). HE staining could be used to assess the degree of tissue damage and tumor type by observing the tissue structure, cell morphology and cell nucleus characteristics. The results of HE staining showed that the tumor tissues of the 5-FU group and the 5-FU + M0-exos group were more vacuolated and had a lighter chromatin staining, which indicated that the 5-FU had a killing effect on the tumor cells, resulting in the loosening of the tumor tissues. In contrast, tumor tissues in the 5-FU + GRP78-exos group were similar to the control group, with dark chromatin staining and more tightly arranged tumor cells (Fig. 3H). Furthermore, the expression of colon cancer stemness markers CD133, CD44, and ALDH1A1 in mouse tumor tissues was detected by immunohistochemistry (IHC) and western blot, and the results showed that there were more CD133, CD44, and ALDH1A1-positive cells in the 5-FU + GRP78-exos group, and the level of their protein expression was markedly elevated (Fig. 3I–K). These results suggest that GRP78-induced macrophage-derived exosomes promote CRC chemoresistance in vivo.

### MiR-769-5p is identified from GRP78-induced macrophages exosomes

**Fig. 4:**
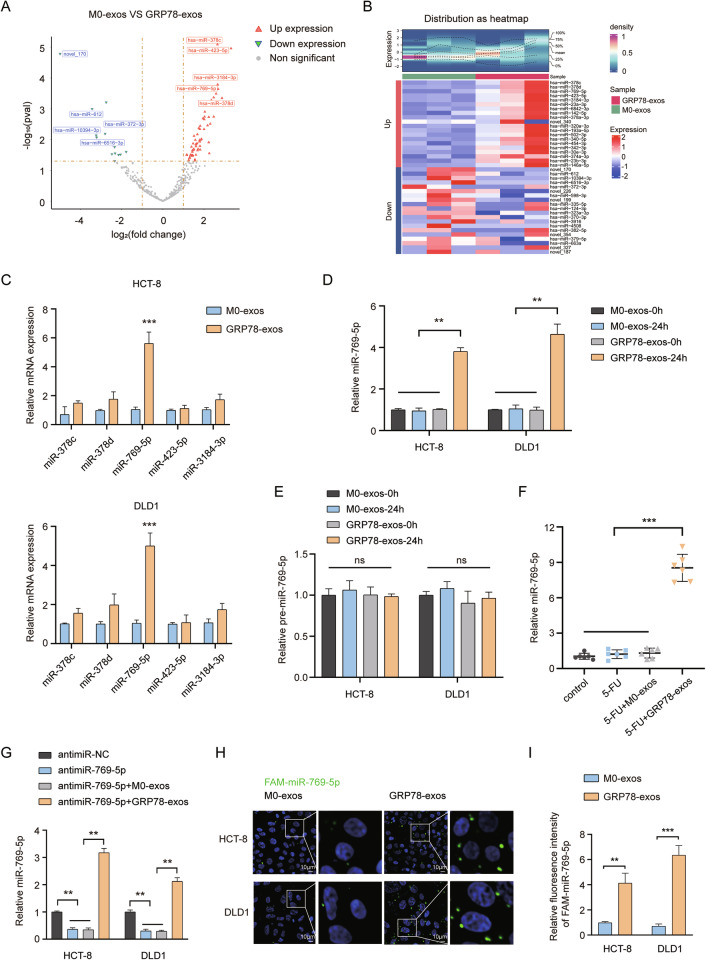
MiR-769-5p transferred directly from GRP78-induced macrophages to CRC cells. **A** A volcano plot of miRNA expression in M0-exos and GRP78-exos. Red color represents miRNAs upregulated and blue color represents miRNAs downregulated in GRP78-exos. **B** Above is a heatmap of data distribution density. Below is the miRNA expression heatmap showing the expression of up-and down-regulated TOP20 miRNAs in the two groups of exosomes. **C** The effects of M0-exos and GRP78-exos on the levels of miR-378c, miR-378d, miR-769-5p, miR-423-5p, miR-3184-3p in HCT-8 and DLD1 cells were detected by qRT-PCR. **D** The effects of M0-exos and GRP78-exos addition for 24 h on miR-769-5p levels in HCT-8 and DLD1 cells were detected by qRT-PCR. **E** The effects of M0-exos and GRP78-exos addition for 24 h on miR-769-5p precursor (pre-miR-769-5p) in HCT-8 and DLD1 cells were detected by qRT-PCR. **F** The levels of miR-769-5p in the serum of each group of mice in Fig. 3 were detected by qRT-PCR. **G** The levels of miR-769-5p in HCT-8 and DLD1 cells after treatment with antimiR-NC, antimiR-769-5p, antimiR-769-5p + M0-exos, and antimiR-769-5p + GRP78-exos were detected by qRT-PCR. **H** M0 macrophage/FAM-miR-769-5p and GRP78 macrophage/FAM-miR-769-5p derived exosomes were co-incubated with HCT-8 and DLD1 cells, and green fluorescence was visualized by fluorescence microscopy. Scale bar: 10 μm. **I** The statistical plot of FAM-miR-769-5p fluorescence intensity in (**H**). Data are expressed as SD ± mean. **p < 0.01, ***p < 0.001. ns no significant difference.

Cell-secreted exosomes can transfer genetic information in the tumor microenvironment and serve as a vital mediator between tumor cells and stromal cell-to-cell communication. Among them, miRNAs are the most representative. Therefore, to further explore the key miRNAs acting in exosomes, we performed miRNA sequencing analysis of M0-exos and GRP78-exos. After differential expression analysis, a total of 39 miRNAs were differentially expressed in the two groups, of which 16 were up-regulated and 23 were down-regulated (Fig. 4A). We also utilized data distribution density heatmap and gene expression heatmap to demonstrate the data availability and up-and down-regulation of TOP20 miRNAs, and the results showed that the data homogeneity in the two groups was better without obvious batch effect, and also visualized the intergroup variation of TOP20 miRNA expression (Fig. 4B). We aimed to find the miRNAs transferred from macrophages to tumor cells, so the top five miRNAs with up-regulated expression in the GRP78-exos group were selected and further verified by qRT-PCR experiments. The results showed that miR-769-5p was most significantly upregulated in CRC cells (Fig. 4C), so in the following experiments, we focused on miR-769-5p.

### MiR-769-5p is directly transferred from macrophages induced by GRP78 to CRC cells via exosomes

To test whether GRP78-exos could increase the level of miR-769-5p in CRC cells, M0-exos or GRP78-exos were co-incubated with CRC cells and the level of miR-769-5p was detected. The qRT-PCR results showed that the level of miR-769-5p in CRC cells significantly increased after adding GRP78-exos for 24 h (Fig. 4D). While the precursor of miR-769-5p (pre-miR-769-5p) was detected under the same conditions and found no significant change in its level (Fig. 4E), these results indicate that the upregulated miR-769-5p in CRC cells is not the result of endogenous synthesis, but is directly transferred by GRP78-exos. In addition, we examined the level of miR-769-5p in the serum of mice in Fig. 3. The results also revealed that miR-769-5p levels were dramatically elevated in the 5-FU + GRP78-exos-treated group (Fig. 4F). To further verify that GRP78-exos could transfer miR-769-5p into CRC cells, we transfected CRC cells with miR-769-5p sponges and then incubated them with M0-exos or GRP78-exos. qRT-PCR results displayed that transfection of miR-769-5p sponges decreased the miR-769-5p levels, but incubation with GRP78-exos significantly increased miR-769-5p levels in CRC cells again (Fig. 4G). After that, we transfected macrophages with fluorescently labeled FAM-miR-769-5p and collected M0-exos and GRP78-exos to co-incubate with CRC cells. The results showed that obvious green fluorescent signal was observed in CRC cells co-incubated with GRP78-exos (Fig. 4H, I). These results suggest that miR-769-5p is directly transferred from GRP78-induced macrophages to CRC cells via exosomes.

### MiR-769-5p derived from GRP78-exos enhances chemotherapy resistance and stemness in CRC cells

**Fig. 5:**
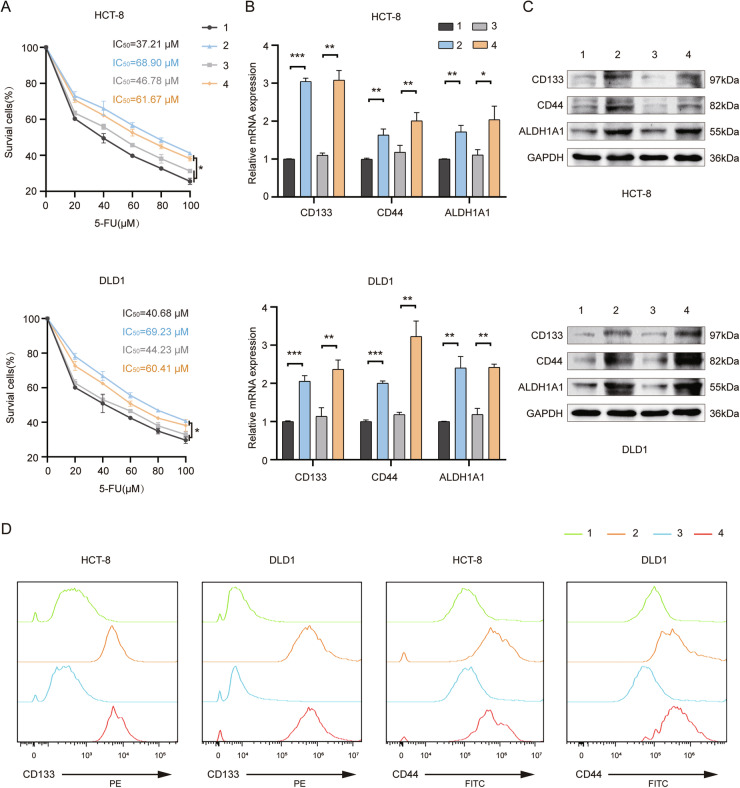
MiR-769-5p derived from GRP78-exos enhances chemotherapy resistance and stemness in CRC cells. HCT-8 and DLD1 cells were treated with 1, 2, 3, and 4. 1: M0-exos. 2: GRP78-exos. 3: GRP78-exos/antimiR-769-5p. 4: GRP78-exos/antimiR-769-5p + miR-769-5p mimics. **A** The effects of the four treatments on cell survival were examined by MTT assay. **B** The effects of four treatments on the mRNA expression of stemness markers CD133, CD44, and ALDH1A1 were examined by qRT-PCR. **C** The effects of four treatments on the protein expression of stemness markers CD133, CD44, and ALDH1A1 were examined by western blot. **D** The effects of the four treatments on the fluorescence intensity of the stemness markers CD133 and CD44 were examined by flow cytometry. Data are expressed as SD ± mean. *p < 0.05, **p < 0.01, ***p < 0.001.

To verify whether miR-769-5p is the key factor in the regulation of drug resistance and stemness in CRC cells by GRP78-exos, the miR-769-5p sponges were transiently transfected in GRP78-induced macrophages, and the exosomes were collected, which is termed GRP78-exos/anti-miR-769-5p, and further the miR-769-5p mimics were transfected is termed GRP78-exos/anti-miR-769-5p + miR-769-5p mimics. The results of MTT showed that the IC_50_ value was significantly lower in the GRP78-exos/anti-miR-769-5p group compared to the GRP78-exos group, while simultaneous transfection of miR-769-5p mimics in CRC cells made miR-769-5p levels elevated and then the IC_50_ value was elevated again (Fig. 5A). The qRT-PCR and western blot results showed that the expression of these stemness markers decreased in the GRP78-exos/anti-miR-769-5p group, while they were upregulated in the GRP78-exos/anti-miR-769-5p + miR-769-5p mimics group (Fig. 5B, C). Similarly, the expression of CD133 and CD44 was greatly reduced in the GRP78-exos/anti-miR-769-5p group, and then enhanced after transfection of miR-769-5p mimics in CRC cells (Fig. 5D). These results indicate that miR-769-5p is a crucial factor in the regulation of CRC cells chemoresistance and stemness by GRP78-exos.

### MiR-769-5p regulates chemotherapy resistance and stemness by targeting MAPK1

**Fig. 6:**
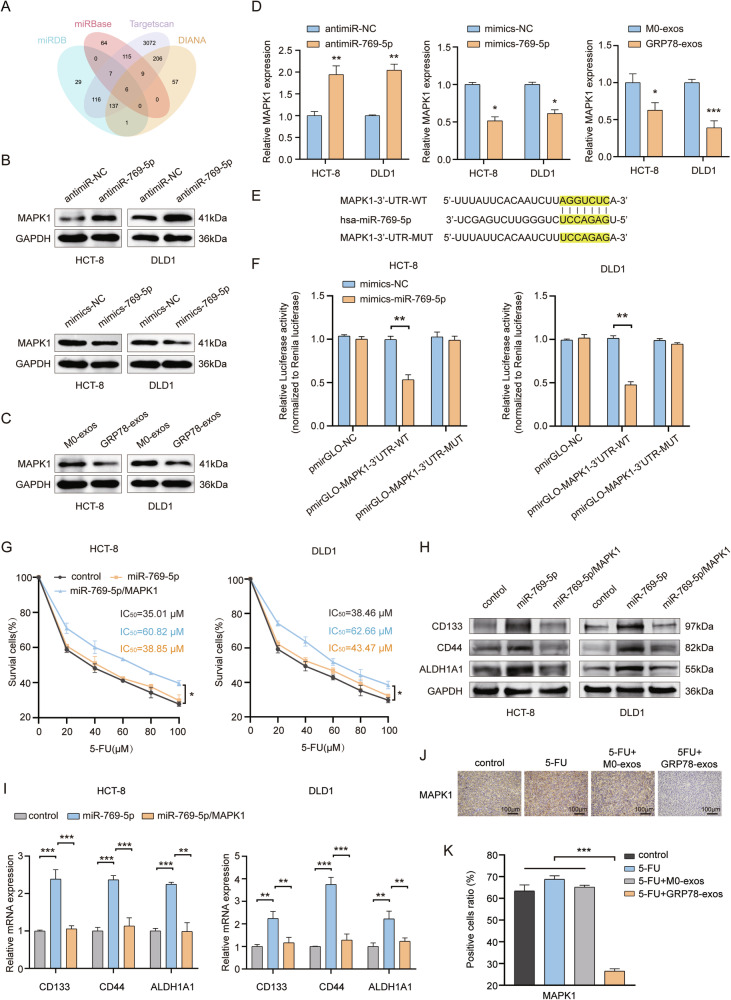
MAPK1 attenuates miR-769-5p-mediated chemotherapy resistance and stemness. **A** A Venn diagram of predicting miR-769-5p target genes using four databases, miRDB, miRBase, TargetScan and DIANA. **B** Protein expression of MAPK1 in antimiR-NC, antimiR-769-5p, mimics-NC, mimics-769-5p-treated HCT-8 and DLD1 cells was detected by western blot. **C** Protein expression of MAPK1 after co-incubation of M0-exos and GRP78-exos with HCT-8 and DLD1 cells was detected by western blot. **D** The mRNA expression of MAPK1 in antimiR-NC, antimiR-769-5p, mimics-NC, mimics-769-5p, M0-exos, and GRP78-exos-treated HCT-8 and DLD1 cells was detected by qRT-PCR. **E** The binding site of the 3’-UTR region of MAPK1 to miR-769-5p was predicted by the TargetScan database and the mutant sequences constructed from the site. **F** The effects of mimics-NC, mimics-miR-769-5p on pmirGLO-NC, pmirGLO-MAPK1-3’-UTR-WT, pmirGLO-MAPK1-3’-UTR-MUT luciferase activities in HCT-8 and DLD1 cells. **G** The effects of transfection of control, miR-769-5p, miR-769-5p/MAPK1 in HCT-8, DLD1 cells on cell survival was detected by MTT assay. **H** The effects of transfection of control, miR-769-5p, and miR-769-5p/MAPK1 on the protein expression of stemness markers CD133, CD44, and ALDH1A1 were detected by western blot in HCT-8 and DLD1 cells. **I** The effects of transfection of control, miR-769-5p, and miR-769-5p/MAPK1 on mRNA expression of stemness markers CD133, CD44, and ALDH1A1 in HCT-8 and DLD1 cells were detected by qRT-PCR. **J** Representative images of MAPK1 immunohistochemical staining of mouse tumor tissue in Fig. 3. Scale bar: 100 μm. **K** Statistical plot of positive cells in immunohistochemical staining for (**J**). Data are expressed as SD ± mean. *p < 0.05, **p < 0.01, ***p < 0.001.

To further explore the downstream targets of miR-769-5p, we screened six downstream targets of miR-769-5p using four bioinformatics and online prediction databases (miRDB [26, 27], miRBase [28–33], Targetscan [34], and DIANA [35]), among which MAPK1 had the highest predictive value and was closely associated with chemoresistance and stemness (Fig. 6A). Next, we transfected miR-769-5p sponges or miR-769-5p mimics in CRC cells. The western blot and qRT-PCR results showed that transfection of miR-769-5p sponges increased MAPK1 expression, while transfection of miR-769-5p mimics decreased MAPK1 expression. This suggests that miR-769-5p is able to negatively regulate MAPK1 expression. Similarly, co-incubation of GRP78-exos with CRC cells also decreased MAPK1 expression (Fig. 6B–D). The Targetscan database predicted the complementary sequence of the 3’-UTR of MAPK1 bound to miR-769-5p. To verify the site-specific inhibition of MAPK1 by miR-769-5p, we constructed wild-type and mutant MAPK1 3’-UTR dual-luciferase reporter genes (Fig. 6E). The results of the luciferase activity assay showed that MAPK1 3’-UTR-WT luciferase activity was significantly suppressed in CRC cells overexpressing miR-769-5p, while MAPK1 3’-UTR-MUT luciferase activity was not obviously changed (Fig. 6F). These results suggest that MAPK1 is the downstream target of miR-769-5p.

We further explored the effect of MAPK1 in miR-769-5p-mediated chemoresistance and stemness enhancement. We constructed MAPK1 overexpression plasmids and transfected them into HCT-8 and DLD1 cells. MTT results showed that the elevated IC_50_ values caused by miR-769-5p were regressed by overexpression of MAPK1 (Fig. 6G). Western blot and qRT-PCR results showed that MAPK1 overexpression notably suppressed miR-769-5p-mediated upregulation of stemness markers (Fig. 6H, I). Additionally, IHC results of mouse tumor tissues in Fig. 3 revealed that MAPK1-positive cells were dramatically reduced in the 5-FU + GRP78-exos group (Fig. 6J, K). The above results indicated that MAPK1 attenuated miR-769-5p-mediated chemoresistance and stemness in CRC cells.

### MiR-769-5p induces CRC cells into quiescence by downregulating MAPK1

**Fig. 7:**
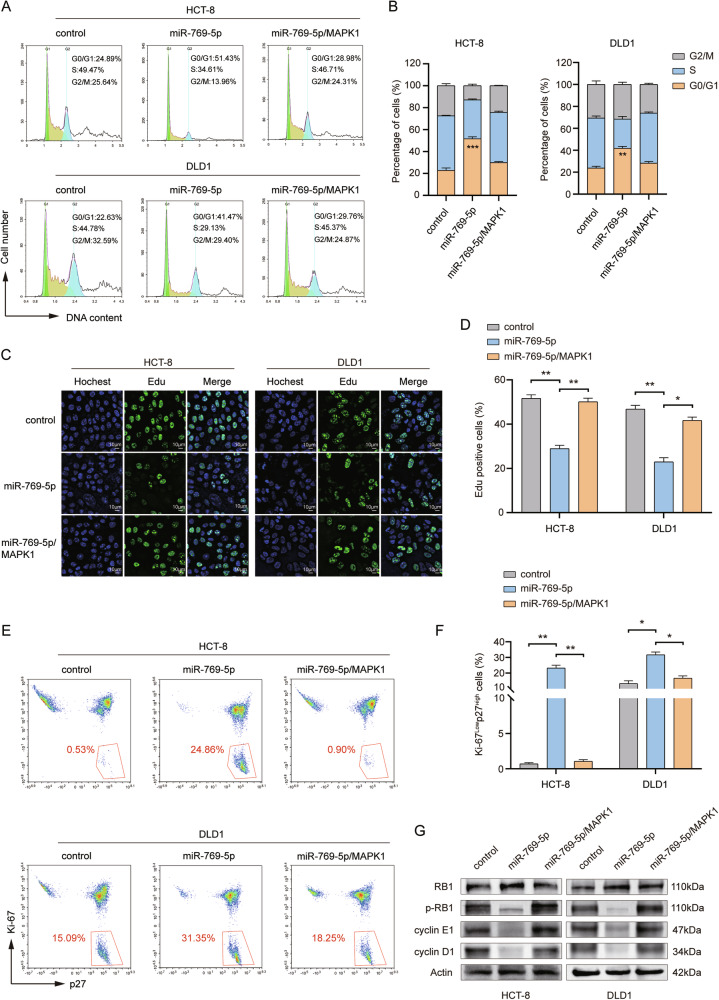
MiR-769-5p induces CRC cells into quiescence by downregulating MAPK1. **A** Schematic representation of the cell cycle in HCT-8, DLD1 cells transfected with control, miR-769-5p, miR-769-5p/MAPK1 as detected by flow cytometry. **B** Statistical plots of the proportion of cells in the G0/G1, S, and G2/M phases for the (**A**). **C** Edu-stained fluorescence images of control, miR-769-5p, miR-769-5p/MAPK1 transfected in HCT-8, DLD1 cells. Scale bar: 10 μm. **D** Statistical plot of Edu positive cells in (**C**). **E** Changes in the ki-67^low^p27^high^ cell population after transfection with control, miR-769-5p, and miR-769-5p/MAPK1 were detected by flow cytometry in HCT-8 and DLD1 cells. Red numbers represent the percentage of this cell population. **F** Statistical plot of the percentage of ki-67^low^p27^high^ cell populations in (**E**). **G** The effects of transfection of control, miR-769-5p, miR-769-5p/MAPK1 on the protein expression of cycle regulatory proteins RB1, p-RB1, cyclin E1, and cyclin D1 in HCT-8 and DLD1 cells were detected by western blot. Data are expressed as SD ± mean. *p < 0.05, **p < 0.01, ***p < 0.001.

MAPK1 is strongly associated with cell cycle regulation [36]. To delve into the mechanism by which MAPK1 attenuates miR-769-5p-mediated stemness and drug resistance in CRC cells, we examined the CRC cell cycle. The results showed that the addition of miR-769-5p resulted in a significant increase of cells in the G0/G1 phase, which was inhibited by MAPK1 overexpression (Fig. 7A, B). Edu staining results also showed that Edu-positive cells were reduced in the miR-769-5p group, and the cell number of this group increased significantly again after MAPK1 overexpression (Fig. 7C, D). This indicated that miR-769-5p blocked the cell cycle of CRC. It has now been shown that tumor cells entering the quiescent phase is an important way to evade chemotherapeutic drugs. Quiescent cancer cells are characterized by low proliferative activity, so ki-67^low^p27^high^ is usually used to represent quiescent cancer cells [37]. Flow cytometry assay showed that the number of cells with ki-67^low^p27^high^ was significantly increased in the miR-769-5p group, and the cell number of this group was significantly decreased again after overexpressing MAPK1 (Fig. 7E, F). Then we detected the expression of G1/S phase-related proteins by western blot. The results showed that the miR-769-5p significantly decreased the phosphorylation level of RB1 and inhibited the expression of cyclin D1 and cyclin E1, which was regressed by overexpressing MAPK1 (Fig. 7G). This suggests that miR-769-5p blocks the cell cycle of CRC cells by down-regulating MAPK1 and induces them to enter the quiescent phase.

### MiR-769-5p is highly expressed in 5-FU-resistant CRC patients

**Fig. 8:**
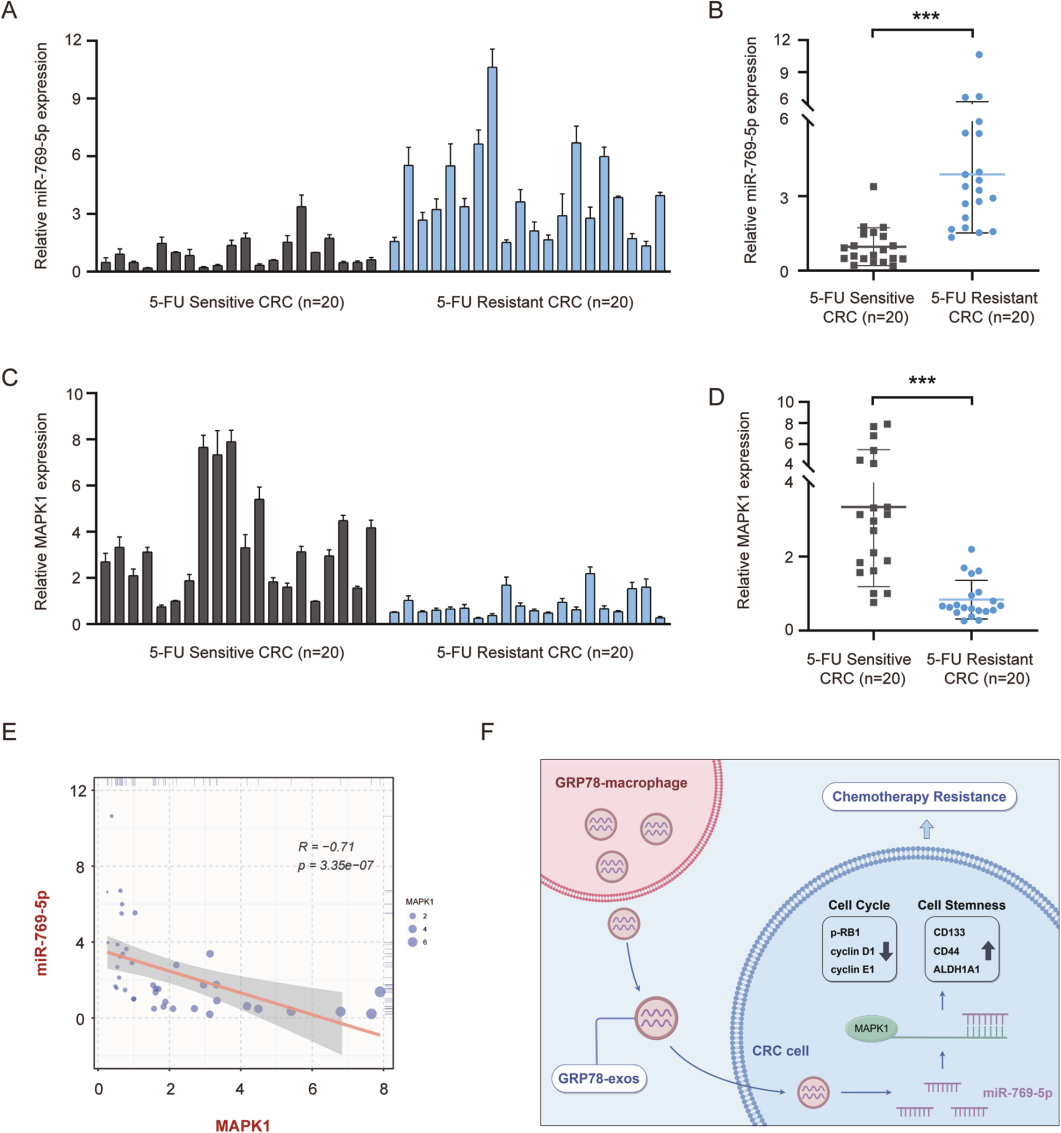
MiR-769-5p is highly expressed in 5-FU-resistant CRC patients. **A** The expression of miR-769-5p in tissues of 5-FU-sensitive (*n* = 20) and 5-FU-resistant (*n* = 20) CRC patients was examined by qRT-PCR. U6 was used as an internal control. **B** Statistical graph of miR-769-5p expression in CRC tissues in (**A**). **C** The expression of MAPK1 in tissues of 5-FU-sensitive (*n* = 20) and 5-FU-resistant (*n* = 20) CRC patients was examined by qRT-PCR. Actin was used as an internal control. **D** Statistical graph of MAPK1 expression in CRC tissues in (**C**). **E** The correlation between miR-769-5p and MAPK1 in CRC tissues was calculated by Spearman’s rank correlation method. **F** Research mechanism diagram by Figdraw. GRP78-induced macrophage-derived exosomes into CRC cells, which contain miR-769-5p targeting MAPK1, inhibited the expression of cell cycle-associated proteins by down-regulating MAPK1 and promoted the expression of stemness markers, leading to CRC resistance.

To validate the clinical value of miR-769-5p in CRC, we detected the expression of miR-769-5p in colon cancer tissues from 40 CRC patients by qRT-PCR. Based on the prognostic model mentioned in Fig. 1, we further constructed a new 5-FU-resistant prognostic model (Fig. S2A–H) by dividing the 40 CRC patient samples into two groups, 5-FU-sensitive and 5-FU-resistant. The results showed that miR-769-5p expression was higher in 5-FU-resistant CRC tissues compared to 5-FU-sensitive samples (Fig. 8A, B). Conversely, MAPK1 expression was lower in 5-FU-resistant samples (Fig. 8C, D). Correlation analysis showed that miR-769-5p was significantly negatively correlated with MAPK1 expression (Fig. 8E). This further demonstrated that MAPK1 was the target gene of miR-769-5p. These results strongly suggest that miR-769-5p may be a predictor of chemoresistance in CRC.

## Discussion

The development of drug resistance is a major cause of colorectal cancer patients’ death. Currently, chemotherapy is still the mainstay treatment for CRC patients, however, most patients can only benefit from chemotherapy in the initial phase due to drug resistance. Therefore, the research on chemotherapy resistance has been gradually deepened in recent years. Some studies have shown that the interaction between tumor cells and stromal cells in the TME can also lead to the development of chemotherapy resistance [38].

The communication between TME and tumor cells is important. As a significant component of TME, macrophages interact with tumor cells and play a crucial role in tumor progression [39]. Tumor cells recruit and domesticate macrophages by secreting a variety of cytokines, which together promote tumor development [40]. After infiltrating into tumor tissues, macrophages also secrete a variety of cytokines and chemokines, forming an immunosuppressive microenvironment that provides favorable conditions for tumor growth [41]. Liu et al. showed that Wnt5a induces macrophage M2 polarization by promoting IL-10 secretion, which in turn promotes CRC growth and metastasis [42]. Zhao et al. demonstrated that the tumor-derived exosome miR-934 induces macrophage M2 polarization and promotes CRC liver metastasis [43]. However, the regulatory mechanism of macrophages in TME and chemoresistance has not been revealed. In this study, we found that GRP78-induced M2-like macrophages promote CRC stemness and chemoresistance via exosomes. This implies to us that the regulation between macrophages and tumor cells is a positive feedback loop in which the two influence each other to promote tumor development. Breaking the balance of this circulation may be a new way of thinking to solve the tumor dilemma.

GRP78 is a molecular chaperone localized in the endoplasmic reticulum and involved in the regulation of calcium homeostasis. A growing number of studies have shown that GRP78 is highly expressed in tumor cells and can be secreted into the TME [44, 45]. Our previous study found that GRP78 secreted by tumor cells recruits and domesticates macrophages in the TME [46], polarizing them to the pro-tumorigenic M2 type [12]. In the current study, we further explored the molecular mechanisms by which macrophages induced by GRP78 promote tumor development in the TME, and identified miR-769-5p with a key regulatory role. As GRP78 is highly expressed in tumors, macrophages induced by GRP78 are also abundantly present in TME. Therefore it is crucial to explore the mechanism of GRP78-induced macrophage promotion of tumor development. It may relatively realistically mimic the stimulation of macrophages in TME, which is important in clinical studies of tumors.

Quiescent cancer cells are cancer cells that have temporarily exited the cell cycle and entered the G0 phase [47]. Quiescent cancer cells can tolerate cell cycle-dependent chemotherapeutic drugs, evade killing by chemotherapeutic drugs, latent in the microenvironment, and then re-enter the cell cycle under specific stimuli, leading to tumor recurrence [48]. A growing number of studies have shown that the ability of CSCs to enter the quiescent state is an important driver of chemotherapeutic resistance [49]. In the study, we found that GRP78-induced M2-like macrophage-derived miR-769-5p caused cell cycle arrest by inhibiting MAPK1 and induced stemness-enhanced CRC cells to enter a quiescent state, which in turn promoted chemoresistance. Therefore, inhibiting CRC cells from entering the quiescent state is one of the effective ways to treat chemoresistance.

Increasing evidence suggests that miRNAs in exosomes play regulatory roles by transferring to receptor cells and binding to corresponding target genes through intercellular communication [50]. It has been reported that miR-769-5p plays a regulatory role in the proliferation, invasion, and metastasis of various cancers [51, 52], but there are few studies on drug resistance. In addition, miRNA is well-suited as a marker for tumor diagnosis due to its high specificity, sensitivity, and stability [53]. Free circulating miRNAs can be detected in serum and other body fluids, thus making sample acquisition and detection relatively easy [54]. In this study, we found that GRP78-induced macrophages transferred miR-769-5p into CRC cells via exosomes, miR-769-5p further inhibited MAPK1 expression by binding to its mRNA 3’-UTR region and promoted CRC stemness and chemoresistance. It was clinically validated that the high expression of miR-769-5p was associated with CRC chemotherapy resistance. This research enriched the regulatory role of miR-769-5p in cancer and identified the potential of miR-769-5p in CRC drug resistance, which suggests that miR-769-5p holds promise as a predictor of chemoresistance in CRC.

## Supplementary information

Supplemental Materials-1

Full length western blots

## Data Availability

All data generated or analyzed during this study are available from the corresponding author on reasonable request.
