# Supplementary material for: MiR-769-5p of macrophage exosomes induced by GRP78 promotes stemness and chemoresistance in colorectal cancer
Source: Cell Death Dis. 2025 Mar 5;16(1):156. doi: 10.1038/s41419-025-07466-7 (PMC11882909; doi:10.1038/s41419-025-07466-7)
Supplement: Supplementary file 1 — Supplemental Materials-1 [file 41419_2025_7466_MOESM1_ESM.docx]

**Figure S1**

**
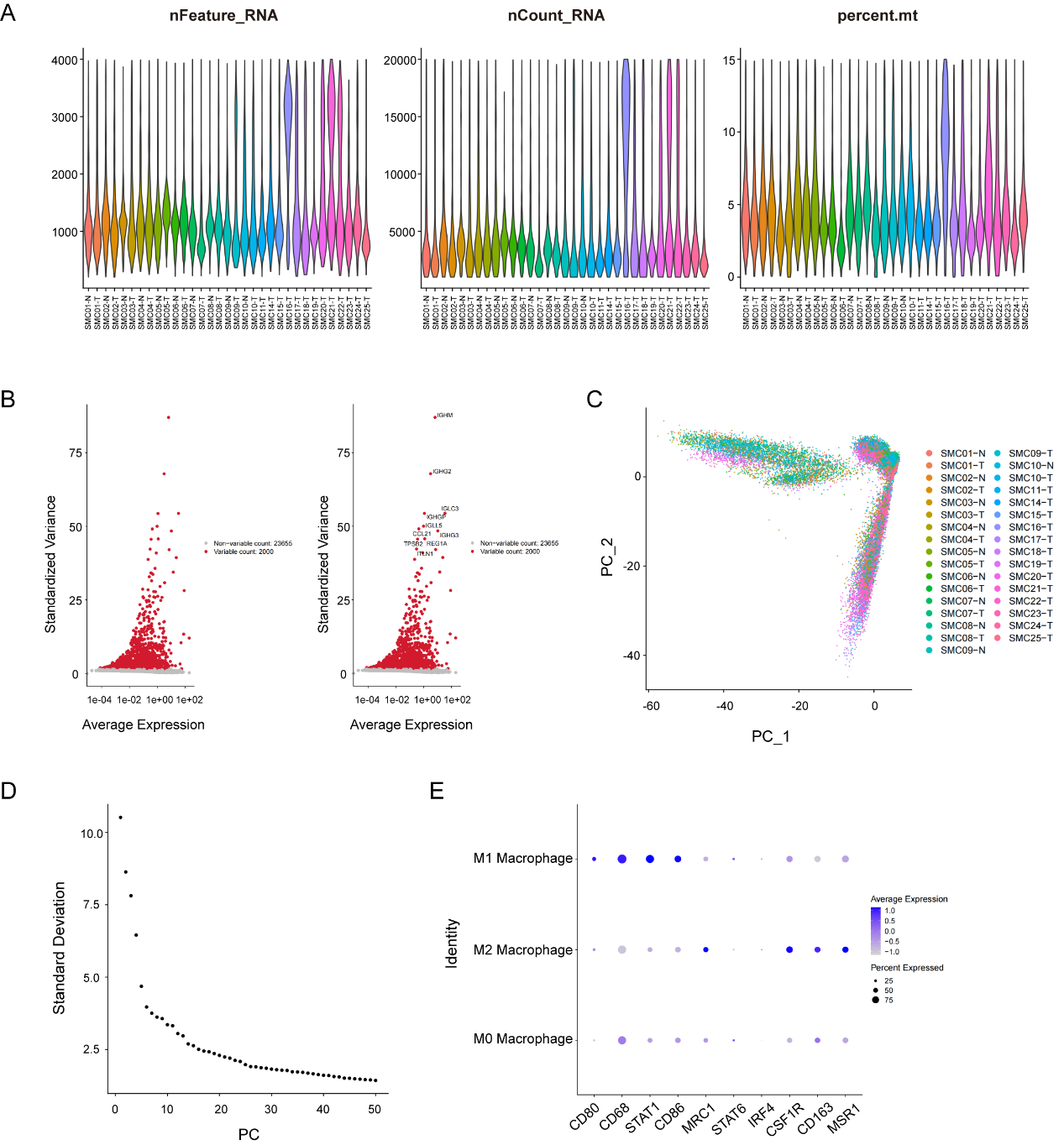
**

**Figure S1. Single-cell sequencing data quality control and standardization.**

A. Violin plots of the distribution of nFeature_RNA (number of genes measured per cell), nCount_RNA (sum of expression of all genes measured per cell) and percent.mt (proportion of mitochondrial genes measured per cell) in the samples after quality control of single cell data.

B. Highly variable genes in single-cell dataset GSE132465. Red color represents highly variable genes. The names of Top10 genes are displayed in the plot.

C. Cell distribution graph obtained from PCA analysis using RunPCA function. The picture shows that the cells are basically uniformly mixed without outliers or abnormal cells, which can be directly analyzed subsequently.

D. The PCA inflection plot plotted for the percentage of information represented by each principal component (percent variance) ranked. Dims = 30 (top 30 principal components) was selected for subsequent analysis.

E. The expression of marker genes after macrophage subsets annotation.

**Figure S2**

**
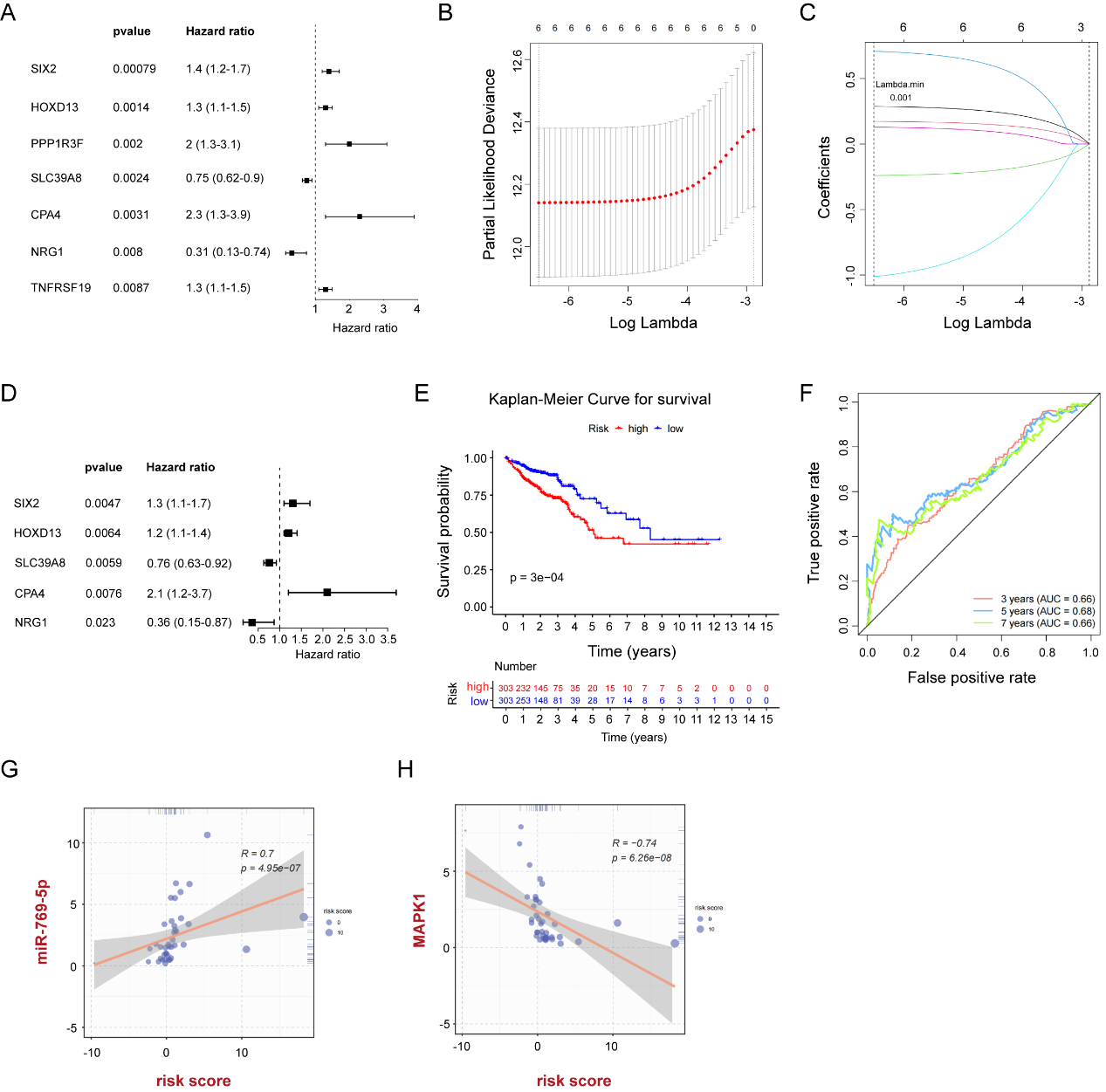
**

**Figure S2. Prognostic modeling based on 5-FU resistance genes.**

1. Univariate COX regression analysis of 5-FU resistance-related genes in TCGA.
2. Partial likelihood deviance of different numbers of variables calculated via the LASSO regression model.
3. LASSO coefficient curves for 5-FU resistance-related genes.
4. Multivariate COX regression analysis of 5-FU resistance-related genes in TCGA.
5. Kaplan-Meier curves for the two groups with high and low risk of 5-FU resistance in the prognostic model.
6. The accuracy of the prognostic characterization was verified by the receiver operating characteristic (ROC) curves and the areas under the curves.
7. The correlation between miR-769-5p and risk score of 5-FU resistance in CRC tissues was calculated by spearman’s rank correlation method.
8. The correlation between MAPK1 and risk score of 5-FU resistance in CRC tissues was calculated by spearman’s rank correlation method.

**Supplementary Materials and Methods**

**Chemical reagents and antibodies**

RPMI-1640 medium was purchased from Pricella (Wuhan, China); Fetal bovine serum (FBS) was purchased from GIBCO (Grand Island, USA); 5-Fluorouracil (Cat. number: F100149, purity≥99%) was obtained from Aladdin (Shanghai, China); PMA (Cat. number: P1585, purity≥99%) and Phalloidine (Cat. number: P5282) were obtained from Sigma-Aldrich (St. Louis, USA); MTT (Cat. number: M8180, purity≥98%) was obtained from Solarbio (Beijing, China); Antibodies for CD133 (Cat. number: bs-0209R), CD44 (Cat. number: bsm-51065M), ALDH1A1 (Cat. number: bs-10162R), TSG101 (Cat. number: bs-1365R), CD63 (Cat. number: bs-1523R), CD81 (Cat. number: bs-6934R), MAPK1(ERK2) (Cat. number: bsm-52068R), KI67 (Cat. number: bs-23103R), p27 (Cat. number: bsm-33425M), GAPDH (Cat. number: bs-41373R), Beta-actin (Cat. number: bs-0061R) were purchased from Bioss (Beijing, China); RB1 (Cat. number: 10048-2-IG), Phospho-RB1 (Cat. number: 30376-1-AP), cyclin D1 (Cat. number: 26939-1-AP), cyclin E1 (Cat. number: 11554-1-AP) were purchased from Proteintech (Wuhan, China); PARP (Cat. number: CY6850), Bcl-2 (Cat. number: CY5582), cleaved Caspase 3 (Cat. number: CY5501) were purchased from Abways (Shanghai, China).

**Cell culture**

Human colon cancer cells HCT-8, DLD1, and human monocyte THP-1 were purchased from the Cell Bank of the Chinese Academy of Sciences (Shanghai, China). The cell lines were all identified using STR analysis. All three cells were cultured in RPMI-1640 complete medium, which was prepared from basal medium with 10% fetal bovine serum and 1% antibiotics.

**Cell survival assay**

Cell viability was detected by MTT assay. Cells in logarithmic growth phase were inoculated in 96-well plates at a concentration of 3,000 per well. After the cells have adhered to the plate, they were treated with M0-CM/GRP78-CM or M0-exos/GRP78-exos for 24 h. Fresh medium was replaced, and 10 μL of MTT was added to each well. After continuing the incubation for 4 h, the old medium was discarded, and 100 μL of DMSO was added to each well, and the plate was shaken at a low speed for 10 min. Absorbance values were measured at 570 nm, and the cell survival rate was calculated.

**Apoptosis assay**

Apoptosis assay was performed according to the method of the kit (Cat. number: C1062S, Beyotime). Cells were digested down with trypsin, washed 2-3 times with PBS and collected. The cells were resuspended by adding 195 μL Annexin V-FITC conjugate to the cell precipitate. Another 5 μL Annexin V-FITC binding solution and 10 μL of PI staining solution were added. The cells were incubated at room temperature for 15 min and detected by flow cytometry.

**Western Blot**

Cells from different treatments were collected, cell lysis solution was added, and cells were lysed by incubation on ice for 30 min. The protein supernatant was collected by centrifugation at 4°C, 12,000×g for 15 min. Protein concentration was determined by BCA method and protein samples with the same content were produced. Electrophoresis was carried out in 10% SDS-PAGE gel at constant pressure, 60 V for the concentrated gel and 90 V for the separator gel. At the end of electrophoresis, the concentrated gel was cut off, and the separator gel was electrotransferred to transfer the protein to the PVDF membrane in a 300 mA constant-current ice-water bath for 2 h. The gel was then blocked with 5% skimmed milk, washed with TBST buffer, and then added to a primary antibody dilution for overnight incubation. The TBST buffer was washed three times, and then the corresponding secondary antibody dilution was added and incubated for 2 h. After washing with TBST buffer, the bands were added dropwise to the TBST buffer. After washing with TBST buffer, chemiluminescent solution was added and the bands were visualized in a protein imager.

**Total RNA extraction and qRT-PCR**

Cells from different treatments were collected, lysed by Trizol method and total RNA was extracted. 500 ng of RNA was taken for reverse transcription to obtain cDNA. cDNA was used as a template for detecting the mRNA expression of the target genes in real-time fluorescence quantitative PCR using SYBR Green Supermix method.

***In vivo* study**

Male Balb/c mice were randomly divided into four groups. 1×10^6^ CT-26 cells in logarithmic growth phase were resuspended in 100 μL saline and injected subcutaneously into mice. When the tumor volume of the mice reached 100 mm^3^, the drug was given. Except for the control group, all three groups were injected intraperitoneally with 20 mg/kg of 5-FU every three days. The control group was replaced with saline. The 5-FU+M0-exos and 5-FU+GRP78-exos groups were simultaneously injected peritumorally with exosomes at 10 μg each time every three days. One month after administration, the mice were executed. Tumor tissues were collected for subsequent studies. The animal experiment was performed according to protocols approved by the Ethics Committee of the Animal Protection Institution of Shanxi University (Protocol Code: SXULL2023043).

Mouse tumor tissues were fixed in formalin and embedded in paraffin. The tumor tissues were stained with hematoxylin-eosin staining for HE staining to observe the structural changes of the tumor tissues. Immunohistochemical staining was performed with antibodies with a dilution ratio of 1:100 to observe the expression of target proteins in mouse tumor tissues. Positive cells were counted with Image J software.

**Luciferase activity assay**

MAPK1-3'-UTR-WT and MAPK1-3'-UTR-MUT fragments were constructed into the pmirGLO vector. HCT-8 and DLD1 cells were inoculated in 24-well plates, and pmirGLO-MAPK1-3'-UTR-WT, pmirGLO-MAPK1-3'-UTR-MUT, or pmirGLO-NC and miR-769-5p mimics were co-transfected into cells, and firefly luciferase and Ranilla luciferase activities were detected 48 h after transfection. The assay was performed according to the steps described in the Dual-Luciferase Reporter Gene Assay Kit (Cat. number: RG027, Beyotime).

**Cell cycle assay**

The experiment was performed using DNA content quantitation Assay (Cell Cycle) kit (Cat. number: CA1510, Solarbio). Cells from different treatments were collected, the cell concentration was adjusted to 1×10^6^/mL, and 1 mL of single-cell suspension was taken. Add 500 μL of 70% pre-cooled ethanol to the cells and fix for 2 h. After washing with PBS, add 100 μL of RNase A solution to the cell precipitates and incubate for 30 min in a 37℃ water bath, then add 400 μL of PI staining solution and incubate for 30 min at 4℃ with light protection, and then record the red fluorescence at the excitation wavelength of 488 nm.

**Edu Staining Experiment**

Edu staining steps were performed as described in the Edu-488 Cell Proliferation Assay Kit (Cat. number: C0071S, Beyotime). After staining was completed, the cells were observed by fluorescence microscopy.
