# Supplementary material for: MiR-769-5p of macrophage exosomes induced by GRP78 promotes stemness and chemoresistance in colorectal cancer
Source: Cell Death Dis. 2025 Mar 5;16(1):156. doi: 10.1038/s41419-025-07466-7 (PMC11882909; doi:10.1038/s41419-025-07466-7)
Supplement: Supplementary file 2 — Full length western blots [file 41419_2025_7466_MOESM2_ESM.docx]

**Full Length western blots**

**Figure 1H**

**
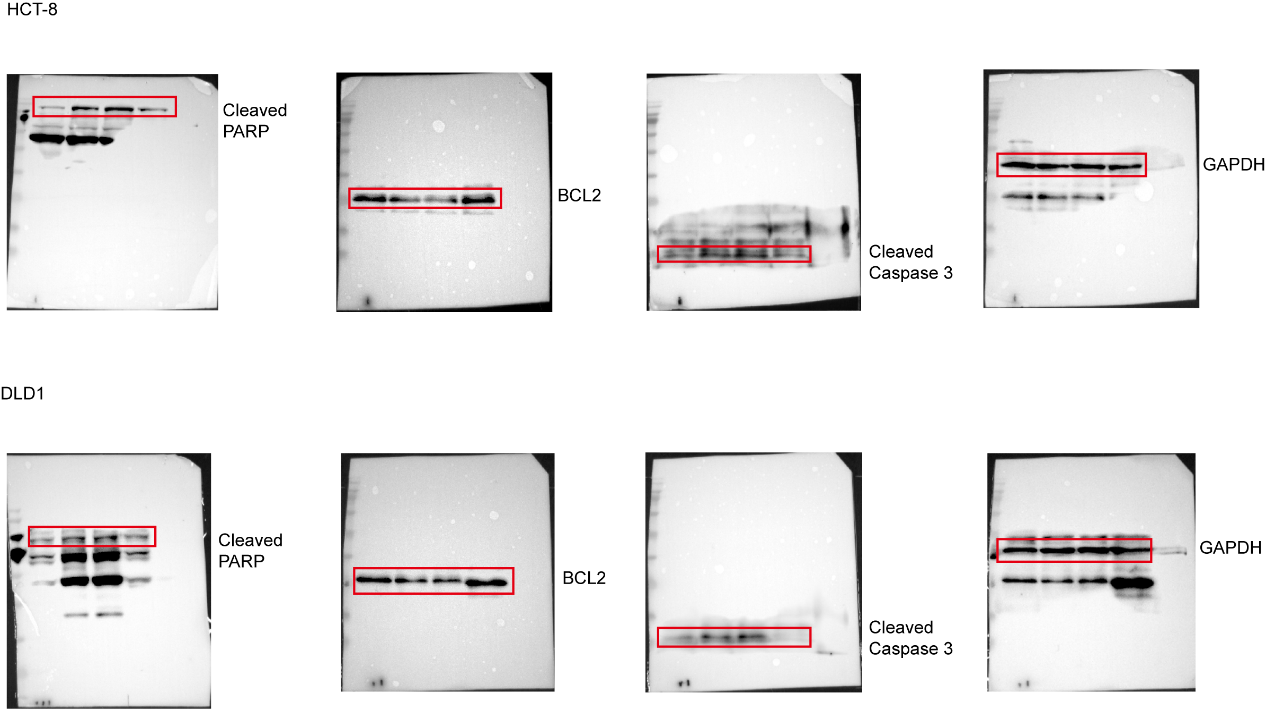
**

**Figure 1I**

**
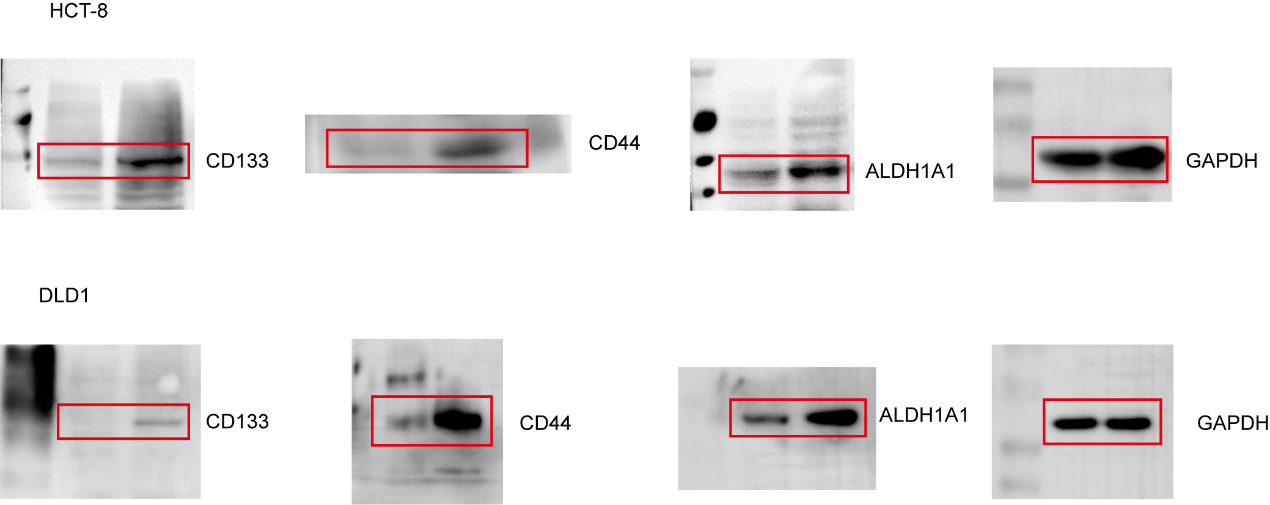
**

**Figure 2C**

**
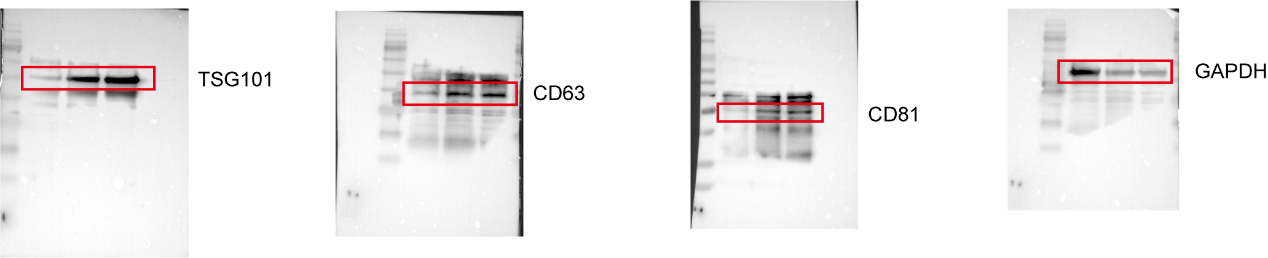
**

**Figure 2H**

**
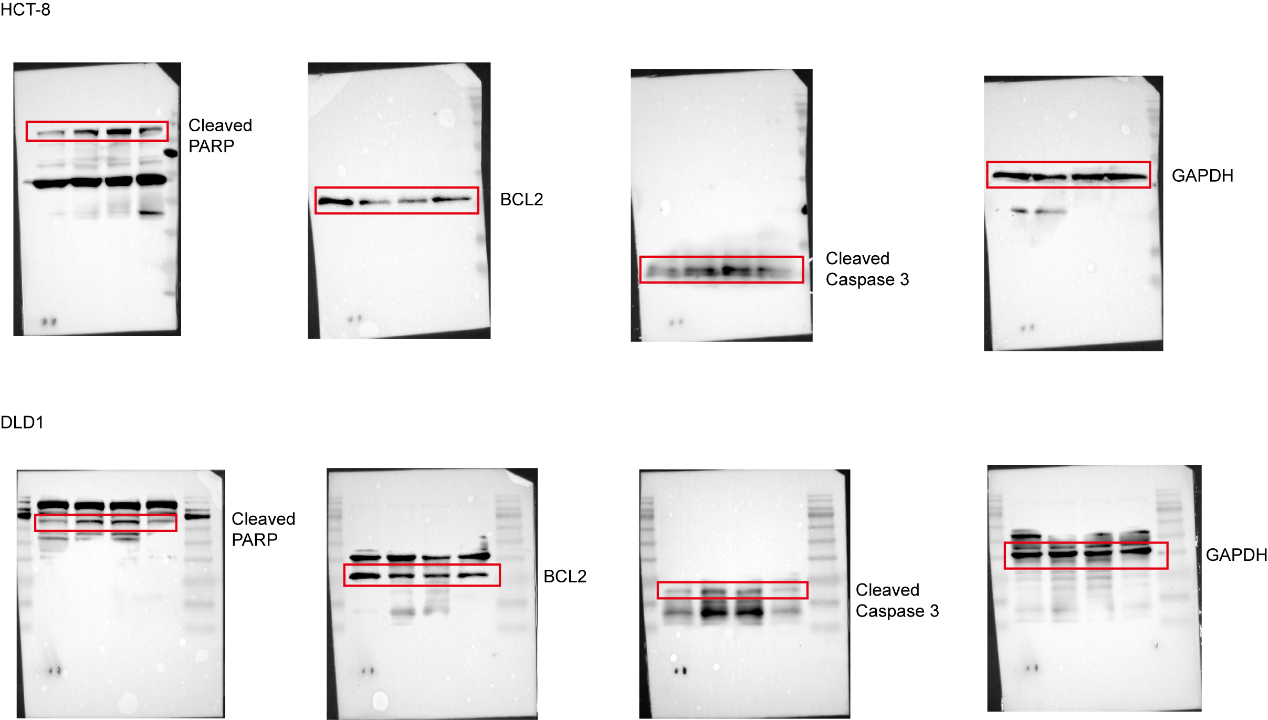
**

**Figure 2I**

**
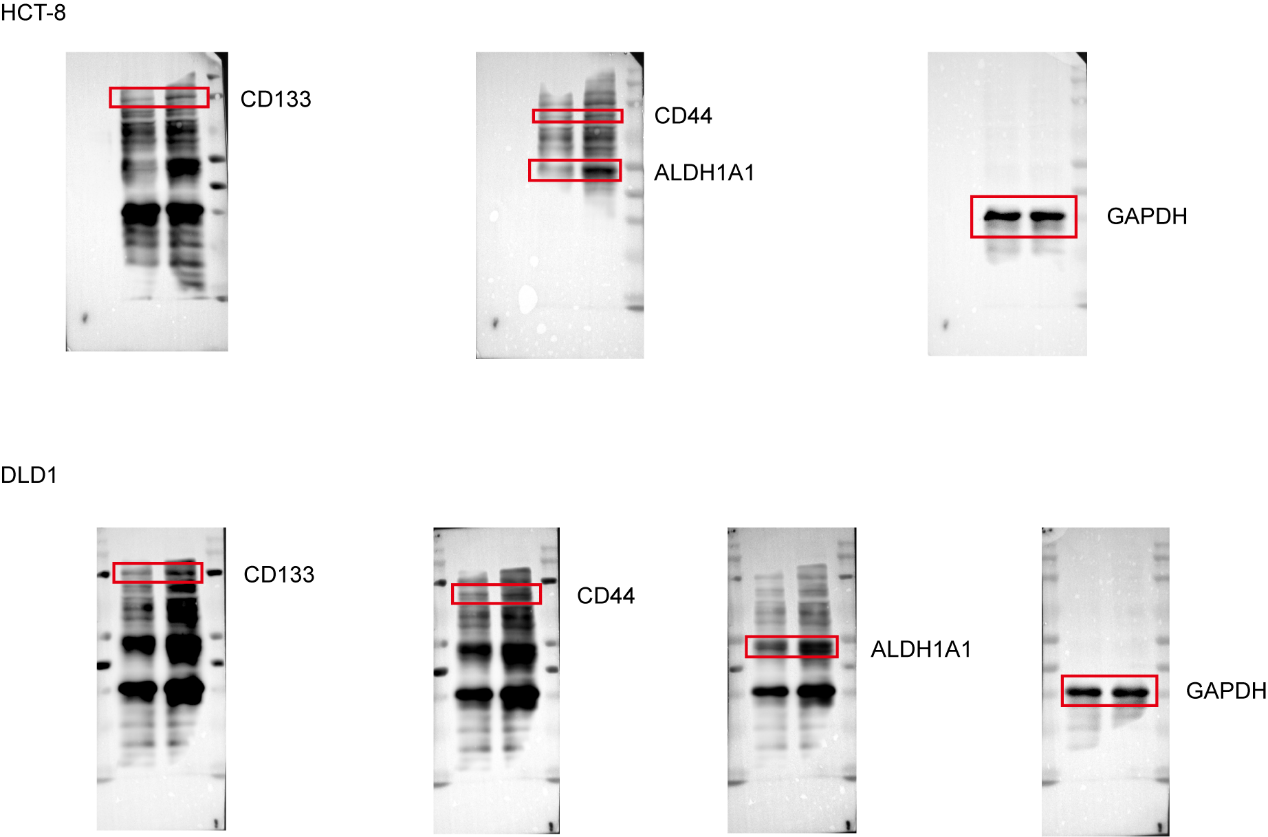
**

**Figure 3K**

**
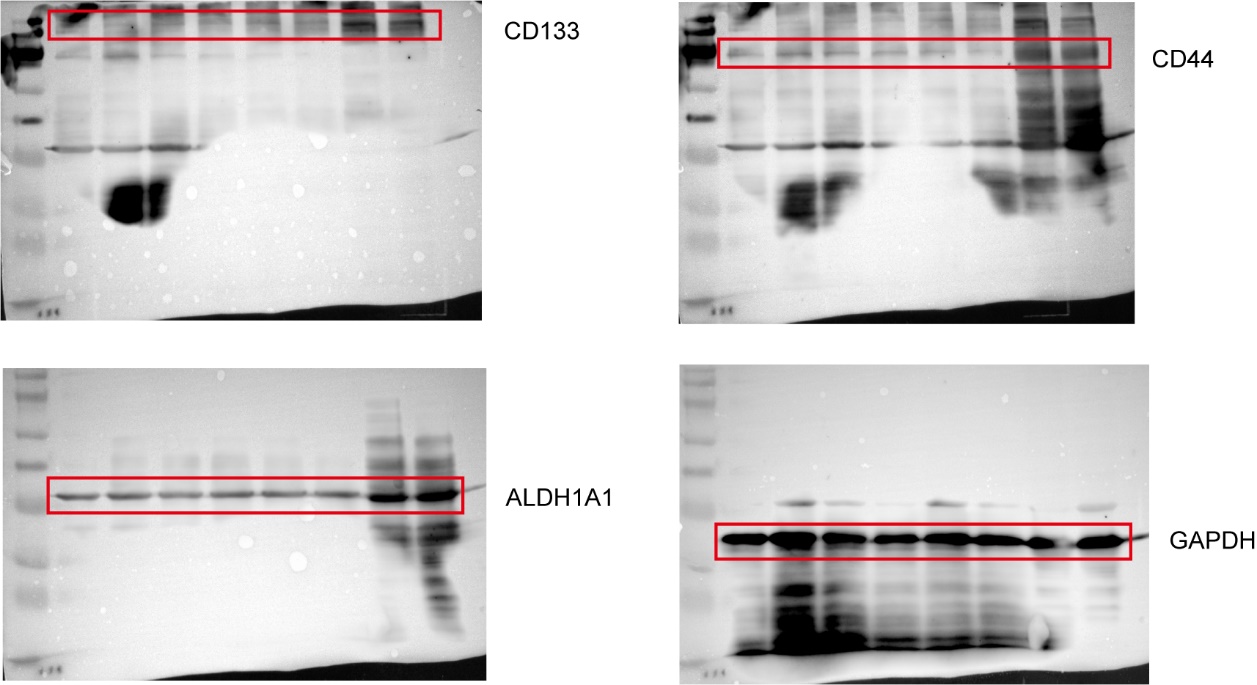
**

**Figure 5C**

**
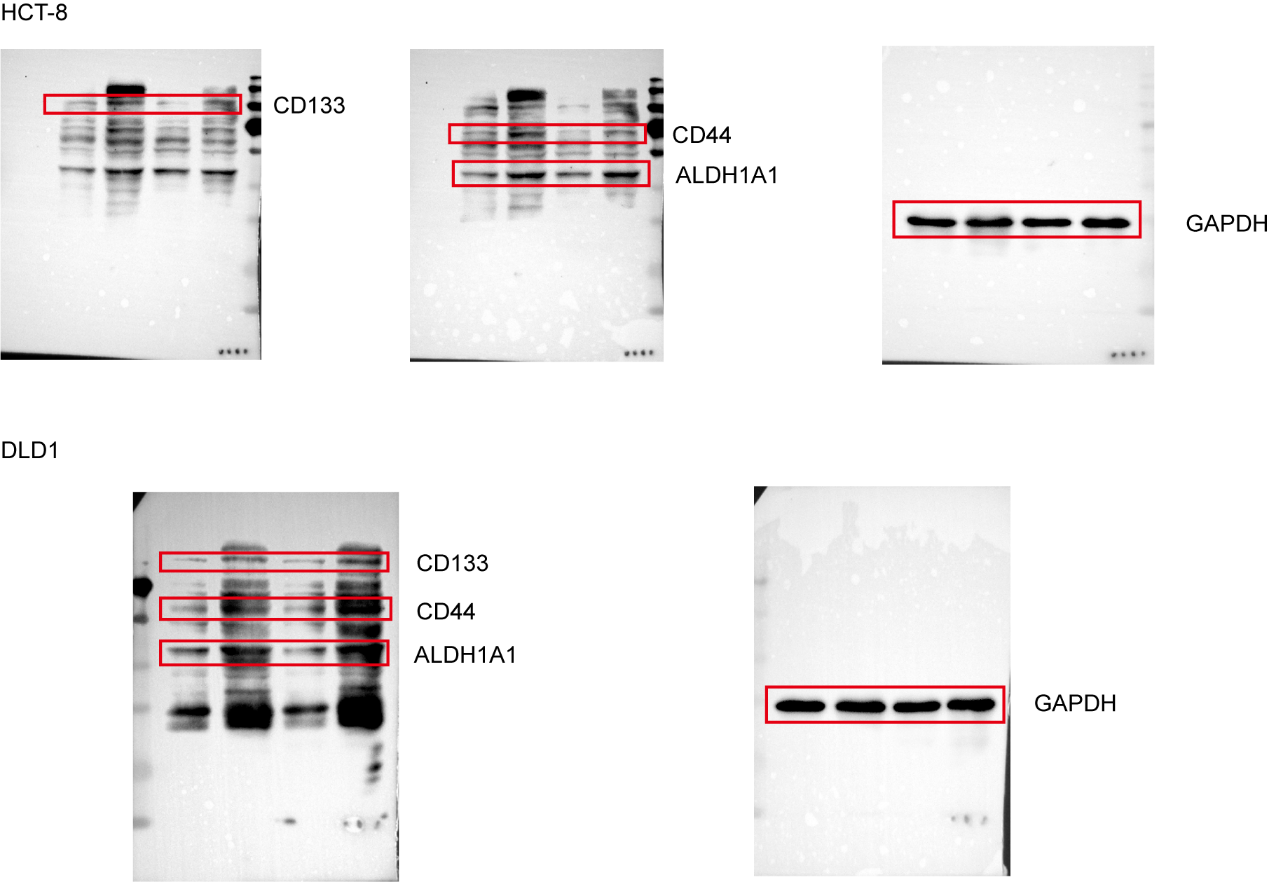
**

**Figure 6B**

**
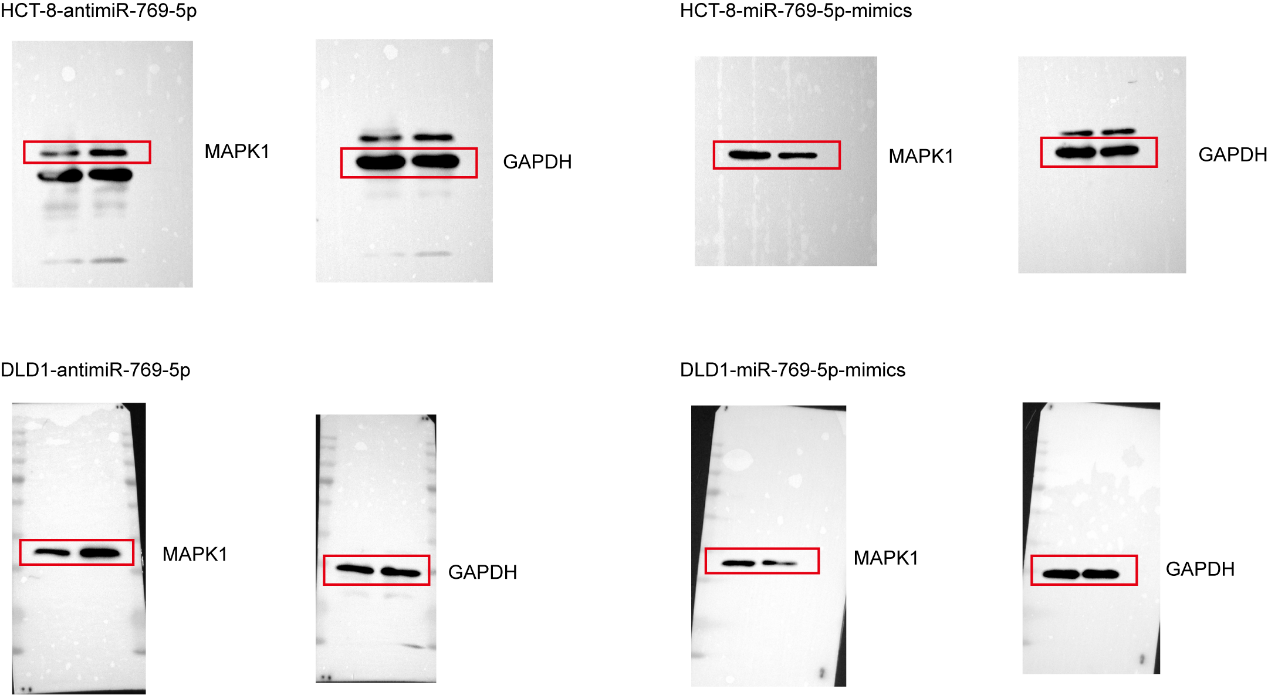
**

**Figure 6C**

**
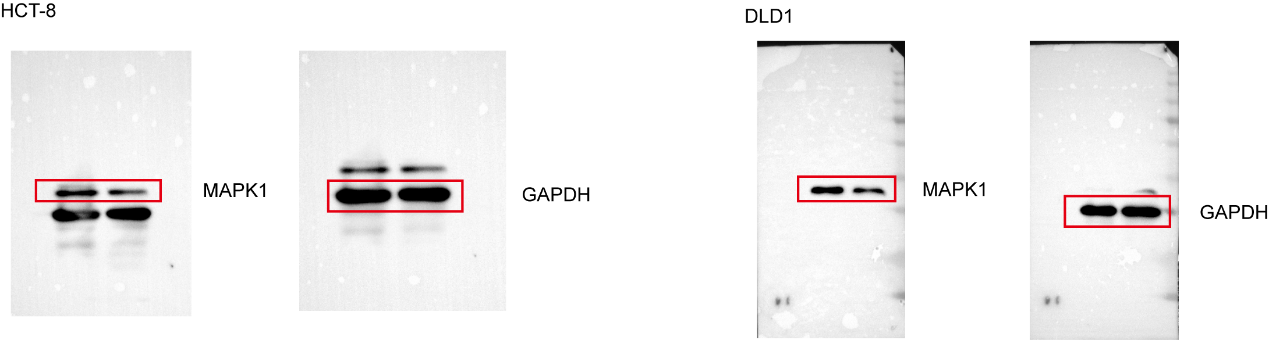
**

**Figure 6H**

**
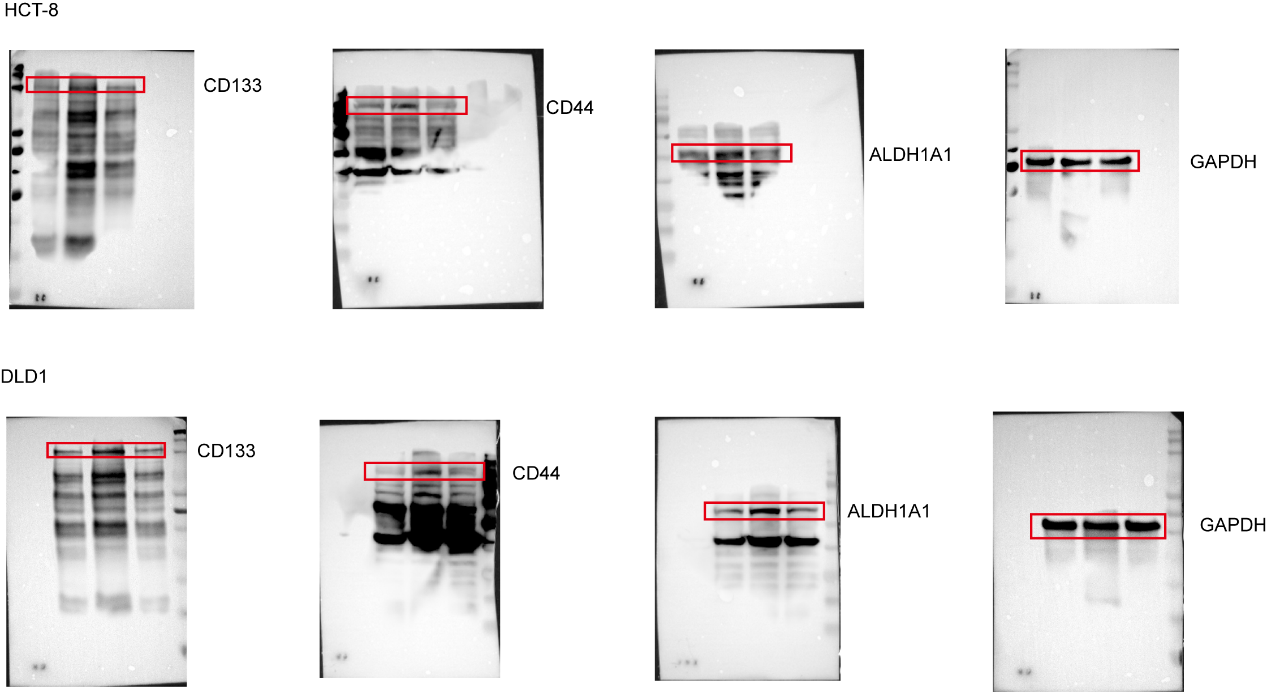
**

**Figure 7G**

**
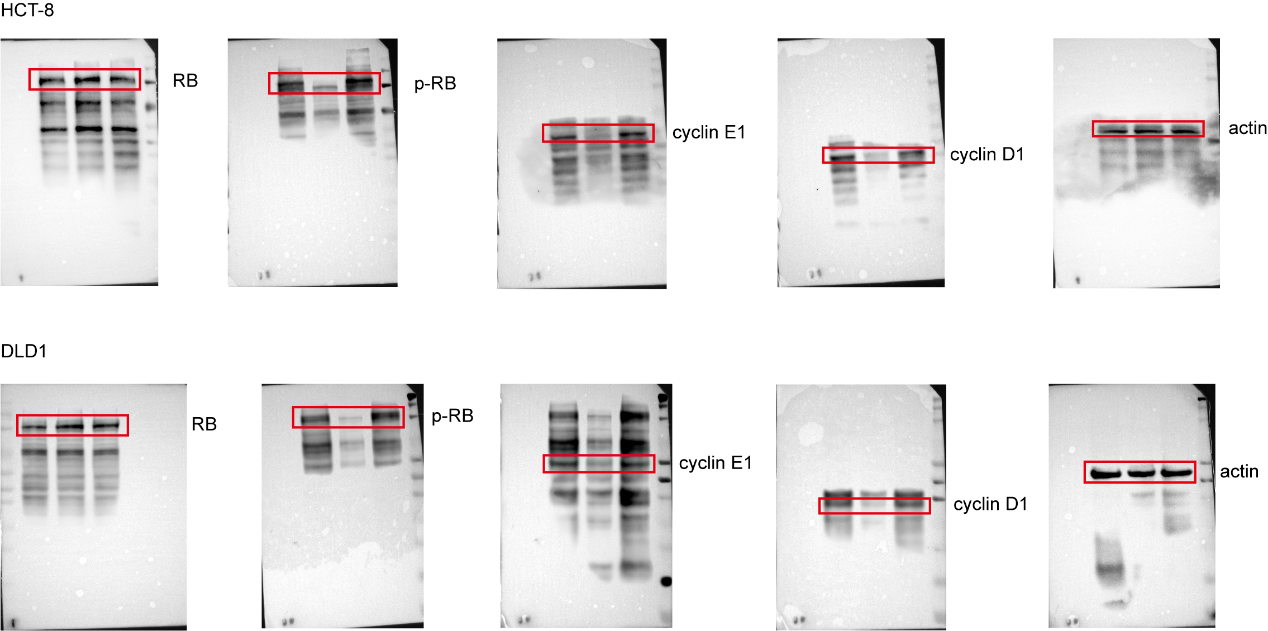
**
